# Supplementary material for: An Online-Based Transition Care Program for Adolescents with Spina Bifida Using Intervention Mapping: A Protocol for Program Development
Source: Int J Environ Res Public Health. 2022 Jan 18;19(3):1056. doi: 10.3390/ijerph19031056 (PMC8834098; doi:10.3390/ijerph19031056)
Supplement: Supplementary file 1 [file ijerph-19-01056-s001.zip › 4. [File S1] mind map.pdf]

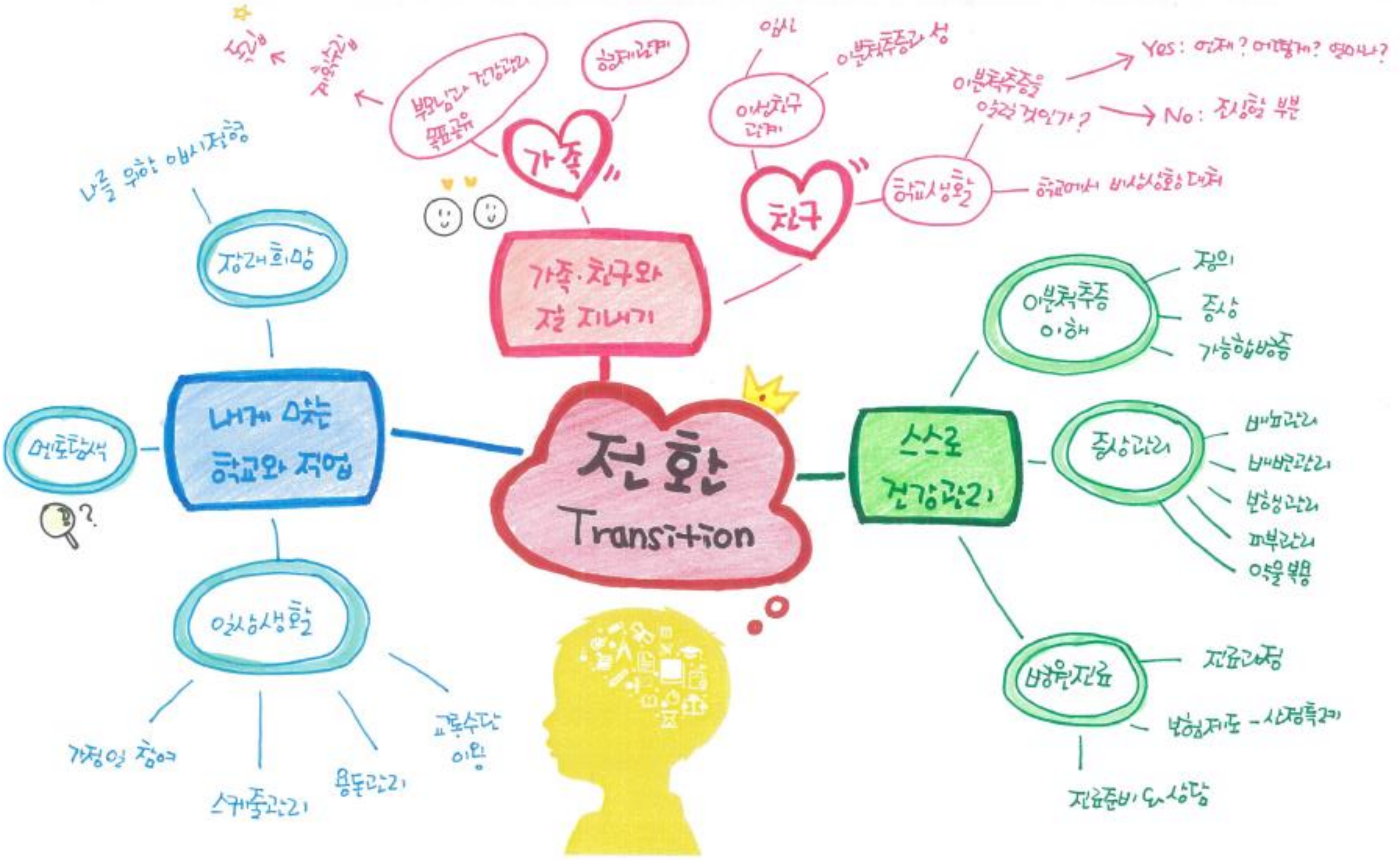

Mind map to express the goals of transition for adolescents with SB; Middle: transition; Left: finding the right school and jobs ; Right: taking care of their own health; Top: getting along well with family and friends.
