# Supplementary material for: An Online-Based Transition Care Program for Adolescents with Spina Bifida Using Intervention Mapping: A Protocol for Program Development
Source: Int J Environ Res Public Health. 2022 Jan 18;19(3):1056. doi: 10.3390/ijerph19031056 (PMC8834098; doi:10.3390/ijerph19031056)
Supplement: Supplementary file 1 [file ijerph-19-01056-s001.zip › 5. [File S2]_ITP.pdf]

Individual Transition Plan (ITP)

• Name: ____________________

• Diagnosis: ____________________

• Date of Birth: ____________________

• Mobile: ____________________

Prioritized goals	Current Status / Plans	Actions	Target date	Complete data	Etc
**[self goal]**<br>**• daily life**					
**• voiding/bowel management**					
**• heallthcare system**<br>(treatment, midicine, appointment)					
**[family goal]**					
**[other goal]**					
**[Memo]**					

• Counsellor: ____________________

• Meeting date: 1st: ____________________

2nd: ____________________
