# Supplementary material for: An Online-Based Transition Care Program for Adolescents with Spina Bifida Using Intervention Mapping: A Protocol for Program Development
Source: Int J Environ Res Public Health. 2022 Jan 18;19(3):1056. doi: 10.3390/ijerph19031056 (PMC8834098; doi:10.3390/ijerph19031056)
Supplement: Supplementary file 1 [file ijerph-19-01056-s001.zip › 6. [File S3] checklist for transition.pdf]

This is what you need to prepare to become an adult. Please "V" check in the place that best describes your current status.

**Transition items**	**Yes**	**No**	**N/A**
1. Healthcare-SB			
1) I know that preparation (transition) is necessary to become a healthy adult.	☐	☐	☐
2) I know my diagnosis.	☐	☐	☐
3) I can explain the physical symptoms caused by spina bifida.	☐	☐	☐
4) I know what to do in case of health-related problems (eg allergic reactions, etc.).	☐	☐	☐
2. Healthcare-voiding management			
5) I purchase and manage catheters (pads) without assistance.	☐	☐	☐
6) I use a catheter (pad) without helps.	☐	☐	☐
7) I know the importance of water intake and the amount of urination.	☐	☐	☐
8) I know the abnormal symptoms of the urinary system (hematuria, fever, pelvic pain, etc.).	☐	☐	☐
3. Healthcare-bowel management			
9) I know the pattern and frequency of my defecation.	☐	☐	☐
10) I cope with fecal incontinence without help.	☐	☐	☐
11) I do enema without help.	☐	☐	☐
12) I know the abnormal symptoms of the bowel problem (blood stool, diarrhea, constipation, etc.).	☐	☐	☐
4. Healthcare-medication			
13) I buy prescription drugs from a pharmacy without assistance.	☐	☐	☐
14) I Know the name, effect, dosage, and precautions of the drugs I take.	☐	☐	☐
15) I Take the medicine accurately without help.	☐	☐	☐
5. Healthcare-neurological health			
16) I know whether or not a shunt is inserted due to my hydrocephalus.	☐	☐	☐
17) I Know the symptoms of shunt dysfunction (headache, vomiting, seizure, etc.).	☐	☐	☐
18) I know the abnormal symptoms of the nervous system (numbness in the senses, weakness in the legs, changes in urine pattern, slurred speech, etc.).	☐	☐	☐
6. Healthcare-orthopedic health			
19) I know the names of assistive devices (braces, crutches, wheelchairs, etc.) used for mobility.	☐	☐	☐
20) I directly manage the assistive devices (orthosis, crutches, wheelchairs, etc.) used to move.	☐	☐	☐
21) I Know the skin abnormalities (redness, swelling, heat, blisters, pressure sores, etc.).	☐	☐	☐

Transition items (cont'd)	Yes	No	N/A
6. Healthcare-orthopedic health (cont'd)			
22) I Knows how to prevent skin abnormalities (check the feet, etc.).	☐	☐	☐
7. healthcare-sexual health			
23) I Knows about general sex education (secondary sexual characteristics, pregnancy, contraception, sexually transmitted diseases, etc.).	☐	☐	☐
24) I know the content of sex education related to spina bifida (sex life problems related to spina bifida, folic acid intake, etc.).	☐	☐	☐
25) I Feel free to discuss with healthcare providers about sexual or pregnancy-related issues.	☐	☐	☐
26) I Feel free to talk with my parents about sexual or pregnancy-related issues.	☐	☐	☐
8. Medical treatment-related issues			
27) I know the health insurance reinforcing policy (system)	☐	☐	☐
28) I know how to issue medical records (medical reports, etc.).	☐	☐	☐
29) I make an appointment for hospital treatment and examination without help.	☐	☐	☐
30) I record or know my treatment and examination schedule.	☐	☐	☐
31) I prepare questions to be asked during treatment in advance.	☐	☐	☐
32) I visit the hospital without help (using public transportation, walking, etc.).	☐	☐	☐
33) I do reception and receipt for hospital treatment and examination without help.	☐	☐	☐
34) I get hospital care and examinations without help.	☐	☐	☐
35) I ask questions during treatment.	☐	☐	☐
9. Daily and school life			
36) I manage my schedule (school and academy schedules, appointments with friends, leisure activities, etc.) without help.	☐	☐	☐
37) I express my thoughts and feelings.	☐	☐	☐
38) I help prepare meals or do it myself.	☐	☐	☐
39) I buy necessary items without help.	☐	☐	☐
40) I use public transportation without help.	☐	☐	☐
41) I receive pocket money and manage the money myself.	☐	☐	☐
42) I explain my health condition to acquaintances or friends.	☐	☐	☐
43) I participate in the Spinal Bifida Patients Association (Kakao Talk, SNS, meetings, etc.).	☐	☐	☐
10. Preparing for the future			
44) I have a dream job for the future.	☐	☐	☐
45) I have a plan to achieve my dream job.	☐	☐	☐

Transition items (cont'd)	Yes	No	N/A
10. Preparing for the future (cont'd)			
46) There is a person I consider to be a mentor.	☐	☐	☐
47) I know the university entrance examination related to spina bifida (equal opportunity admission- persons with special conditions).	☐	☐	☐
11. Family			
48) My family allows me to do it myself.	☐	☐	☐
49) I share and discuss my future with my family.	☐	☐	☐
50) I discuss my health condition with my family.	☐	☐	☐
